# Geospatial data on the sediments of Lake Balaton

**DOI:** 10.1038/s41597-024-02936-7

**Published:** 2024-01-18

**Authors:** Mihály Kocsis, László Pásztor, András Makó, Piroska Kassai, Kálmán Csermák, Alice Csermák, Erzsébet Aradvári-Tóth, Gábor Szatmári

**Affiliations:** 1 Institute for Soil Sciences, HUN-REN Centre for Agricultural and Environmental Research, Budapest, Hungary; 2 National Laboratory for Water Science and Water Security, Budapest, Hungary

**Keywords:** Limnology, Hydrology

## Abstract

Freshwater lakes in most inhabited areas of the world are threatened by water quality issues. Standard water conservation measures have shown efficiency in the past; however, polluted lakes have only partially recovered from eutrophication. Our knowledge is still incomplete about the sensitivity of these lakes to different anthropogenic sources and to the changes in their internal processes due to global warming. In this article, we present a database comprising sediment data from Lake Balaton (Hungary), which can facilitate further analysis helping to better understand the internal processes and changes occurring in the lake. The published dataset includes the following parameters measured in 4211 lake bed sediment samples: pH_KCl_, calcium carbonate (CaCO_3_), organic carbon (C_org_), total nitrogen (N_total_), soluble phosphorus (AL-P_2_O_5_) and soluble potassium (AL-K_2_O), magnesium (Mg^2+^), zinc (Zn^2+^), copper (Cu^2+^) and manganese (Mn^2+^). We are confident that our database serves as a strong basis for further research relating to freshwater lakes influenced by human activities.

## Background & Summary

Lake Balaton is the largest freshwater lake in Central Europe. It is considered not only one of Hungary’s most important natural treasures, but also a highly popular tourist destination with significant economic value. Throughout recent history, there have been major changes in the land use around the lake (urbanisation, railway construction, over-use of surrounding land for agriculture), which have had a significant impact on the extent of the water surface and the position of the lake shore. Unfortunately, these changes have led to the ecological degradation of the lake since the mid-20th century. In the 1970s and 1980s, the lake underwent a rapid eutrophication episode, causing a dramatic decline in water quality^1^. The main cause of eutrophication was the loss of function of the natural filtration/buffer area (now called Kis-Balaton) at the estuary of the lake’s largest inflowing river (the Zala River) due to the drop in water levels. Unfortunately, the intensive, yield-oriented agricultural production during the socialist era further exacerbated nutrient overload and by the 1980s it had reached critical levels.

In the following decades, targeted environmental measures (reduction of external phosphorus load and restoration of Kis-Balaton) have halted eutrophication, leading to a substantial improvement in the ecological status of the lake^2–4^. However, despite all the previous efforts to protect water quality, significant algal blooms have been observed again in recent years. Istvánovics *et al.*^5^ found that the recurrence of algal bloom events is most likely related to global warming, leading to significant changes in the internal lake processes. Numerous scientists believe that a warming climate affects and modifies the thermal structure of shallow lakes and reduces mixing by increasing the thermal stability of the water column^6,7^. This can lead to a rapid hypolimnetic near-bottom oxygen deficiency, which increases the release of phosphorus (P) from the sediments^8,9^. In the case of Lake Balaton, external load regulation is no longer sufficient to control eutrophication^5^. Since the internal P load is derived from the lake bed, it is very important to obtain and evaluate all available information on the physical and chemical properties of the lake bed sediments to better understand this process.

As sampling and assessment of lake sediments is usually very expensive and labour intensive, detailed surveys are rarely carried out. As climate change intensifies, legacy data can be extremely valuable in comprehending the underlying processes in our changing environment. A positive trend to collect data from previous surveys into open databases and making it accessible for further research, is a response to this demand. There are already numerous open databases created for this purpose. For example, the PLUTO geochemical database contains more than 120,000 records of sediments (lakes, streams and other sediments) collected and analysed by the United States Geological Survey from across the United States^10^. Some states (Louisiana, Minnesota, or Wisconsin) have their own databases specifically for lake sediment data. Canada also has an open database^11^ available for research that preserves historical and current sediment data, remediation, lake and shoreline management, habitat studies, and engineering projects. VARDA (Varved Sediments Database)^12^ provides data specifically on annually laminated lake sediments from around the world. This database allows comparison of well-dated and annually laminated sediment records to reconstruct abrupt and regional temporal changes.

Currently, no common open database exists for lake sediments in the European Union, but some Member States have their own platform. No such database has been available in Hungary, despite the recurrent ecological problems associated with Lake Balaton. Although detailed research on the physicochemical properties of the sediments of Lake Balaton was carried out in the 1970s and 80 s, at which time interval more than 5,000 samples were collected, it was only accessible to a narrow professional circle. Several results have already been published from the analysis of the database^13,14^, yet the full potential inherent in its use has not been fully realized. So, together with the authors of the survey, we decided to publish this dataset in the form of a digital, open access database. The aim of this study is to present this database and make it widely available for future use both nationally and internationally. On the Zenodo^15^ platform, the following parameters are available from the original survey of Lake Balaton sediments: pH_KCl_, CaCO_3_, C_org_, N_total_, AL-P_2_O_5_, AL-K_2_O, Mg, Zn, Cu, Mn.

Potential users of this database are professionals, including hydrologists and limnologists, who are studying the effects of land use and climate change on freshwater lakes to prevent further ecological disasters.

## Methods

### Sampling strategy

The sampling strategy and laboratory methods of the Lake Balaton sediment survey are summarized based on the work of Csermák & Máté^16^.

**Fig. 1:**
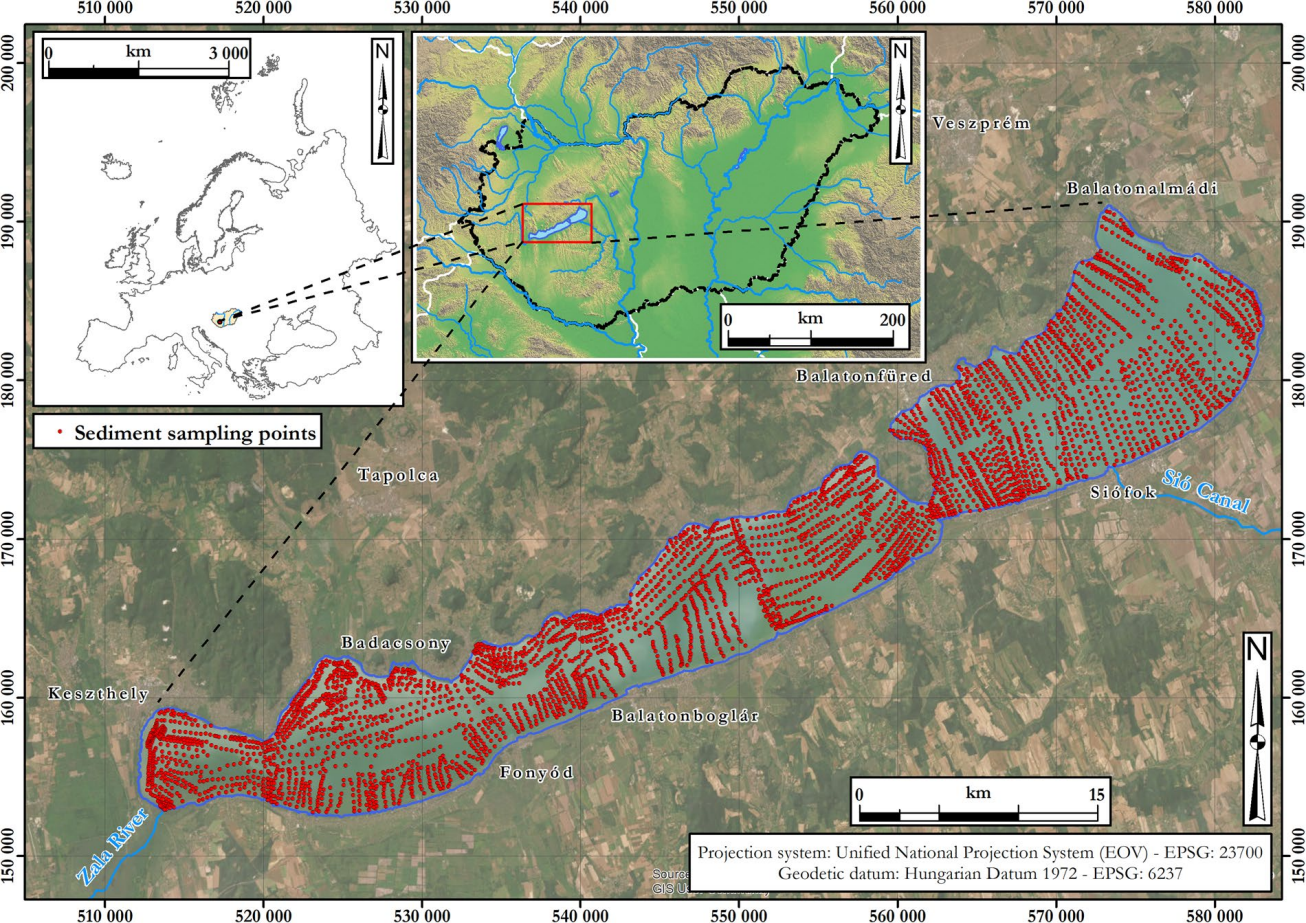
Spatial location of Lake Balaton and the 4211 sample points of the sediment survey.

The sample density was adjusted to the intended cartographic scale of the planned survey maps, i.e., 1:10,000, which is the same scale as the topographic maps used to identify the lake shore. Based on their soil survey experience, Csermák & Máté^16^ found, that mapping on this scale requires about 10 sediment samples per km^2^. As the sediment was more homogeneous spatially in some areas than expected, the survey team worked with a density of 7–10 samples per km^2^ in the less disturbed inner area of the lake. Altogether, 4211 sediment samples were collected from a total of 592 km^2^ of water-covered area (Fig. 1). The vertical sampling design took into account that the top 10 cm layer of sediment plays a more important role in water quality compared to the deeper layers. Since the vertical inhomogeneity of the upper sediment layers may not be significant due to regular mixing and bioturbation, mixed sediment samples were taken from the upper 10 cm layer (without more accurate depth information).

Sampling was carried out at higher spatial frequencies at natural and artificial water inlets and near wastewater treatment plants to detect local anomalies in sediment parameters due to possible contamination. Most samples were taken between 1978 and 1984 in May and August. The latest samples from the lake shore region were collected in three distinct areas and times: the sediments of the reedbeds in 1985–1986, the inflow estuaries in 1987–88, and the area around the sewage treatment plants in 1989. Sampling was carried out with an Ekman-Birge sediment sampler^17^. However, a simple spoon sampler was also used at some sampling points, as the Ekman-Birge grab was impractical for harder sediments.

### Laboratory analyses

The following parameters were examined from the sediment samples in the laboratory: pH_KCl_, hydrolytic acidity (hy_1_), calcium carbonate (CaCO_3_), organic carbon (C_org_), total nitrogen (N_total_), soluble phosphorus (AL-P_2_O_5_) and soluble potassium (AL-K_2_O), magnesium (Mg^2+^), zinc (Zn^2+^), copper (Cu^2+^), manganese (Mn^2+^), sodium (Na^+^), sulphate (SO_4_^2−^).

Due to the large number of samples, costly and labour-intensive procedures could not be applied. The analyses were carried out in the soil laboratories of the Plant Protection and Agrochemical Stations of Fejér and Zala County (Hungary) using well-established and partially automated methods of soil testing practices. Ammonium-lactate (AL) extractant was used for sample pre-treatment for nutrient content measurements (phosphorus, potassium) as it dissolves the plant-available forms. All test methods (Table 1.) are still commonly used in Hungary as these measures are included in the national standards.

**Table 1:** Summary of laboratory test methods applied to the sediment samples.

Parameter	Method	Unit
Organic carbon (C_org_)	Tyurin method^19^	%
pH_KCl_	Potenciometer	-
Calcium carbonate (CaCO_3_)	Scheibler’s calcimeter	%
Hydrolytic acidity (hy_1_)	Kappen’s method^20^	-
Total nitrogen (N_total_)	Kjeldahl method^21^	%
Soluble phosphorus (AL-P_2_O_5_)	AL extract, atomic adsorption spectrophotometry^22^	mg kg^−1^
Soluble potassium (AL-K_2_O)	AL extract, flame photometer^22^	mg kg^−1^
Magnesium (Mg^2+^)	KCl extract, atomic adsorption spectrophotometry^23^	mg kg^−1^
Zinc (Zn^2+^)	EDTA + KCl extract, atomic adsorption spectrophotometry^24^	mg kg^−1^
Copper (Cu^2+^)	EDTA + KCl extract, atomic adsorption spectrophotometry^24^	mg kg^−1^
Manganese (Mn^2+^)	EDTA + KCl extract, atomic adsorption spectrophotometry^24^	mg kg^−1^
Sodium (Na^+^)	AL extract, flame photometer^22^	mg kg^−1^
Sulphate (SO_4_^2−^)	KCl extract, atomic adsorption spectrophotometry^25^	mg kg^−1^

### Geospatial (GIS) processing

In the early 2000s, the GIS Laboratory of the Research Institute for Soil Science and Agricultural Chemistry, Hungarian Academy of Sciences (RISSAC HAS) digitally processed all sample sites and the related laboratory test results and organized them into a geospatial database. This information had previously been recorded on paper-based maps and protocols. Data from 4211 samples were included in a vector-based point database, from which preliminary digital sediment maps were generated using distance-based buffering (Thiessen polygon formation method) and ordinary kriging for the spatial extension of the measured chemical parameters^16^.

## Data Records

The dataset^15^ is available on the Zenodo online repository at 10.5281/zenodo.10076359.

The full dataset is organized in a single CSV file that also contains the X Y coordinates defining the spatial location of the sample points. In the published database there are only 10 parameters out of the measured 13 due to significant data gaps. We also performed filtering on the data, which is described in detail in the Technical validation section. The final number of the samples of the published 10 parameters can be seen in Table 2. The coordinate system of the original map was converted from Hungarian Datum 1972 into WGS 1984 in the published database, but we also kept the original (HD 72) coordinates.

**Table 2:** Number of samples by parameters in the Lake Balaton sediment database.

Parameters	Number of samples
Point identification	4 101
Sample identification	4 101
WGS 1984 X coordinate	4 101
WGS 1984 Y coordinate	4 101
HD 1972 X coordinate	4 101
HD 1972 Y coordinate	4 101
Organic carbon (C_org_)	3 606
pH_KCl_	3 776
Calcium carbonate (CaCO_3_)	2 867
Total nitrogen (N_total_)	4 030
Soluble phosphorus (AL-P_2_O_5_)	3 340
Soluble potassium (AL-K_2_O)	3 665
Magnesium (Mg^2+^)	3 963
Zinc (Zn^2+^)	3 852
Copper (Cu^2+^)	3 994
Manganese (Mn^2+^)	3 973

Missing values are replaced with ‘−2’ in the database for easier identification.

## Technical Validation

The laboratory tests were carried out in accredited soil laboratories (Plant Protection and Agrochemical Stations of Fejér and Zala County) following the current Hungarian standards. After the vectorization of the data points, we filtered the dataset in two steps: first, we identified data records resulting from digitization errors (e.g., mistyping) and removed them from the dataset. Secondly, we identified data records affected by measurement error using a spatial filtering approach, as follows: (i) we computed the averages of all 10 parameters for data points within 1 km of each other, (ii) we generated raster layers (of all parameters) with the calculated averages, (iii) we determined a threshold for each parameter from the difference between the measured parameter and the computed mean value based on descriptive statistics and distribution curves, and (iv) we identified and removed the data points where the deviation between the measured and the calculated parameter was larger than the threshold. The maximum allowable deviation (threshold value) was 0.25% for organic carbon content, 0.50% for pH_KCl_, 5% for calcium carbonate content, 20 mg kg^−1^ for soluble phosphorus (AL-P_2_O_5_) content, 0.50 mg kg^−1^ for total nitrogen content, 100 mg kg^−1^ for soluble potassium (AL-K_2_0) content, 500 mg kg^−1^ for magnesium content, 3 mg kg^−1^ for zinc content, 5 mg kg^−1^ for copper content, and 30 mg kg^−1^ for manganese content. With this filtering procedure, the incorrect values resulting from sampling/measurement errors were removed.

An important question that needs to be addressed is to what extent the values of the measured parameters may have changed since the original survey. A few years after the original survey a resampling was carried out on some sample points and the results showed minimal percentage point deviations compared to the original measurements^16^. However, a number of water protection actions were implemented after the survey, which improved the quality of the settling sediment. As the sediment accumulation rate is 7–10 mm/year^18^, the new fresh sediment on the top layers most likely less polluted. Nevertheless, the internal physical factors (flow conditions, location of inlets) are unchanged, so it can be assumed that although the individual parameters can change over time (e.g., less phosphorus arrives), the spatial pattern remains the same. In addition, the database provides accurate information on the nutrient distribution of past sediment layers that currently positioned in deeper layers, but can be important, e.g., in predicting the environmental effects of planned dredging or other actions affecting the lake bed.

## Data Availability

No custom code was used in creating the dataset.
